# The Efficacy of HIIT Programs for the Improvement of Aerobic Capacity and Functionality for Stroke Survivors: Systematic Review and Meta-Analysis

**DOI:** 10.1155/nri/4543683

**Published:** 2025-10-20

**Authors:** Elena Papamichael, Irene-Chrysovalanto Themistocleous, Stelios Hadjisavvas, Demetris Solou, Christina Michailidou

**Affiliations:** ^1^Department of Health Sciences, School of Life and Health Sciences, University of Nicosia, Nicosia 2417, Cyprus; ^2^Department Klinik für Neurologie und Neurophysiologie, Universitatsklinikum Freiburg, Freiburg 79106, Germany

**Keywords:** aerobic function, gait, HIIT, motor function, physiotherapy, stroke

## Abstract

Cerebrovascular accident is a neurological disease, characterised by acute onset that lasts for more than 24 h, leading to motor, sensory and cognitive impairments or even death. High-intensity interval training is a type of aerobic training that presents an increase of the > 80% of maximum heart rate, aiming to improve VO_2_ peak, leading to improvements in various health-related parameters. The purpose of this study was to examine the effectiveness of high-intensity interval training on aerobic and functional capacity for poststroke survivors. Two investigators searched the electronic databases MEDLINE/PUBMED, Cochrane Controlled Trials Register and EBSCO, until August 2024. In this review, 11 studies met the eligible criteria and were included. The statistical analysis was conducted by pooling the mean, standard deviation, and 95% confidence intervals. For the establishment of meta-analysis, the heterogeneity statistical index *I*^2^ was used. From the 11 included studies, 458 stroke survivors were extracted. HIIT yield improvements were observed in VO_2_ peak (*p* value = 0.001, 95% CI: 1.72–4.06), 6MWT (*p* value < 0.001, 95% CI = 38.55–149.41), 10MWT (*p* value < 0.01, 95% CI = 0.20–0.36), BBS (*p* value < 0.01, 95% CI = 3.43–7.51), EQ-5D (*p* value = 0.001, 95% CI = 3.67–15.13), and cognition (*p* value = 0.009, 95% CI = 0.41–2.89). No significant difference was presented for HR (*p* value = 0.58, 95% CI = −11.82–21.10), TUG (*p* value = 0.055, 95% CI = −2.25 to 0.02) and step count (*p* value = 0.71, 95% CI = −1479–2163). High-intensity interval training is a safe rehabilitation method affecting positively the aerobic capacity and the majority of motor function of stroke survivors.

## 1. Introduction

Cerebrovascular attack (CVA) or stroke is a neurological disease of vascular origin of the central nervous system (CNS), characterised by acute onset that lasts more than 24 h leading to motor, sensory, and cognitive impairments or death [1, 2]. Based on the aetiology, stroke is classified into two main categories, haemorrhagic stroke (HS) and the ischaemic stroke (IS). The main difference between HS and IS depends on their cause. IS, resulting from cerebral infarction after thrombotic or embolic attack, that limited the blood flow of a cerebral area. IS is the most common type (87%) of CVA, presenting a large proportion of disability worldwide [3]. It occurs due to the reduction of blood into the vessels of the brain after the presence of a thrombotic or embolic event. Thrombotic attack appears as a result of arterial dissection, atherosclerosis or inflammation [4]. On the other hand, embolic attack is caused by atherosclerotic plaques in proximal arteries presenting different types like cardioembolism, lacunar infarcts and large artery atherosclerosis [4, 5]. On the other hand, HS includes intracerebral and/or subarachnoid haemorrhage, due to rupture of blood vessels, provoking internal bleeding into the brain parenchyma or into the subarachnoid space [6, 7]. The aetiology of HS includes hypertension, cerebral amyloid angiopathy, myocardial infarctions, cerebral artery dissection and aneurysm rupture [2, 7]. Further risk factors that can lead to CVA include cardiovascular disorders, obesity, smoking, high blood cholesterol, hypertension, diabetes mellitus, smoking, stress, chronic liver disease, age, gender, limited physical activity and nonhealthy nutrition behaviours [5, 7–9].

Stroke is the third leading cause of disability and the second leading cause of death in the world [10]. The incidence of stroke globally in 2022 was 12.2 million new cases, with an overall of 101 million people who experienced stroke [10, 11]. The majority of patients with stroke are older people of more than 70 years (67.3%), whereas younger people (15–49 years old) present a percentage of 22.4%, which is an increase of incidence by 15% between 1990 and 2019. High incidence of stroke is related to gender, that affects more women (54.9%) in comparison to men [11]. The American Heart Association (AHA) in 2024 reported an increase of 26.3% in death rate following stroke (162.890 deaths) in the United States for the decade of 2011–2021, whereas in 2021, the worldwide mortality was 7.44 million people (50.5% with HS) [12]. Apart of the high death rates, stroke also results in psychomotor limitations leading to disabilities, presenting an increase of 32% on disability-adjusted life years (DALY) in comparison to 1990 [10].

Stroke covers a spectrum of various neurological symptoms and conditions including motor (e.g. hemiparesis, hemiplegia, weakness, spasticity, balance disruption and gait deficits), nonmotor symptoms (e.g. aphasia, cognitive impairments, agnosia and sensory deficits) [12–14], cardiovascular and metabolic changes (e.g. decreased daily metabolic expenditure, decrease blood flow in affected leg, expiratory muscle dysfunction causing decrease in cardiorespiratory fitness and high fatigue) [15, 16] and psychological disorders (e.g. mood changes, depression, anxiety and apathy [17, 18]. For the recovery of poststroke patients, the evaluation of functional parameters is essential. More specifically, the assessment of aerobic capacity, motor and cognitive function, as well as the evaluation of quality of life (QOL) are necessary for assessing the impact of stroke, leading to the development of an interventional program [19, 20].

Guidelines for CVA rehabilitation recommend using moderate intensity of continuous exercise (MICE) for the improvement of cardiorespiratory fitness [21]. However, the intensity in these programs is not high enough to reach significant improvements [22], and as a result, MICE is not commonly used in clinical settings [23]. On the other hand, higher exercise intensity programs with controlled duration and frequency lead to better functional parameters [24]. High-intensity interval training (HIIT) is a type of aerobic training that provides high exercise enjoyment level with low-intensity recovery periods and shorter duration of exercises, consisting of increased heart rate (HR) (> 80% of maximum HR), aiming to improve VO_2_ peak [25, 26]. More specifically, HIIT can be categorized into long-interval HIIT (for 3–4 min, with exercise:rest ratio 1:1 or 4:3), short interval HIIT (100%–120% VO_2_ peak, 15–60 s with exercise:rest ratio 1:1) and the low-volume HIIT (near-maximal or maximal absolute or anaerobic workload, for 10–60 s with exercise:rest ratio 1:2 or 1:4). In general, the suggested frequency for improving stroke health–related outcomes was 2–3 times per week for 4 weeks in total [27]. HIIT seems to improve the oxidative capacity of skeletal muscle through increase neuromuscular recruitment, leading to better functional capacity in nondisabled population [28]. Despite the commonly use of HIIT in rehabilitation program for different populations with improvements in various health–related parameters [29–31], its use in poststroke survivors is narrow, maybe because of the presence of muscle function reduction, motor and nonmotor limitations that can result in a decrease of exercise tolerance leading to physical inactivity and deterioration of health level [15, 32], activities of daily living (ADL) and reduction in QOL [33].

The purpose of this systematic review and meta-analysis was to collect, examine and provide evidence-based information regarding the application of HIIT programs for the aerobic capacity, motor and cognitive functions for poststroke individuals.

## 2. Methods

**Figure 1:**
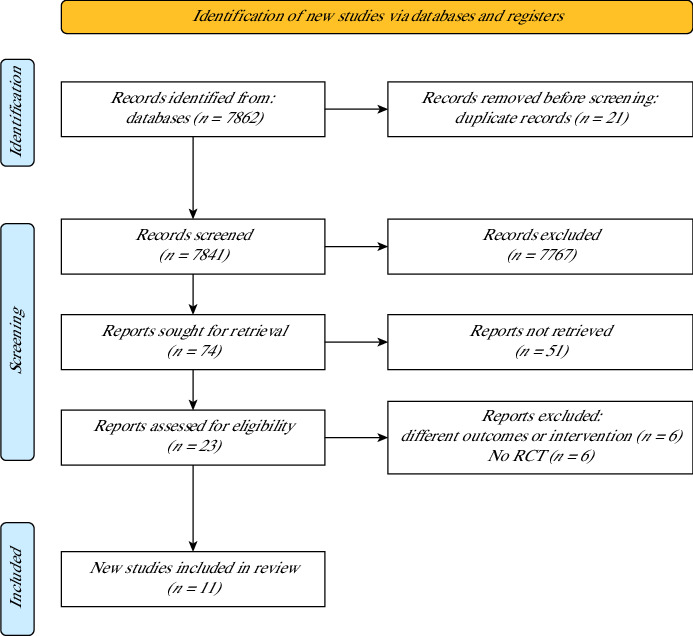
Flow diagram of the literature search procedure.

The current systematic review was registered in PROSPERO database (CRD42024568535), followed the Preferred Reporting Items for Systematic reviews and Meta-Analyses (PRISMA) statement (guidelines 2020) and applied the population, intervention, control and outcomes (PICO) approach. For this systematic review, the studies were published in 2014–2024 in the English language. Therefore, the following electronic databases were searched in July and August 2024: Medical Literature Analysis and Retrieval System Online (MEDLINE/PUBMED), Cochrane Controlled Trials Register (CENTRAL/CCRT) and EBSCO.  Figure 1 shows the searching and screening process followed.

### 2.1. Inclusion Criteria

The keywords that were used for the search were ‘Cerebrovascular accident' OR ‘CVA' OR Stroke AND ‘High intensity interval training' OR ‘HIIT' AND ‘Aerobic training' OR ‘Traditional Physiotherapy' OR ‘Physiotherapy' AND ‘Aerobic function' OR ‘Motor Function' OR ‘Gait' OR ‘function'.

The inclusion criteria used were: (1) randomized control trials (RCTs), (2) stroke diagnosis (ischaemic and/or haemorrhagic), (3) HIIT as experimental intervention (involving any assistive equipment like canes), (4) other training or educational aerobic programs (as a comparison group), (5) assessment of aerobic capacity (VO_2_ peak), physical function (motor, gait, balance), cognitive function and physiological parameters (HR).

### 2.2. Quality Assessment of Studies

**Figure 2:**
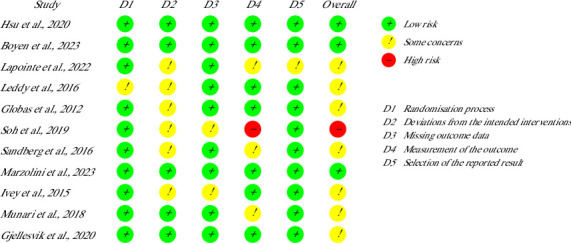
Risk of bias assessment.

The evaluation of the studies was conducted in two phases, in phase 1: reviewer Elena Papamichael used the keywords to search for papers, screened the titles and abstracts of the identified papers, and in phase 2: reviewer Irene-Chrysovalanto Themistocleous checked the full text of the eligible studies. If any discrepancy appeared, a third reviewer, Demetris Solou, resolved the differences. For the data extraction, the reviewers Elena Papamichael, Irene-Chrysovalanto Themistocleous and Stelios Hadjisavvas independently extracted critical data from each selected study, using a customized Excel sheet with the application of PICOT framework. The Cochrane risk of bias tool (RoB2) was used by two reviewers (Elena Papamichael and Irene-Chrysovalanto Themistocleous) to assess risk of bias. The reviewer Christina Michailidou resolved any disagreement between individuals' judgement. Risk of bias results are presented in Figure 2.

### 2.3. Data Synthesis and Analysis

The data synthesis and analysis were conducted by researchers EP and DS. The statistical software SPSS 29.00 was used for statistical analysis, finding the postintervention mean, standard deviation (SD) and confidence interval (CI). The statistically significant difference level was set at < 0.05. To examine eligibility for meta-analysis, homogeneity evaluation was conducted via the Levene's test, where the Sig. Index was set at > 0.05. In meta-analysis, the interventions of the control group were grouped together (including aerobic and educational aerobic training) and compared with the experimental group (HIIT programs). OpenMeta-analyst software was used for meta-analysis, where the continuous random-effects DerSimonian and Laird models with 95% CI were applied. For the identification of heterogeneity, the index *I*^2^ with significant level > 75% was applied. Moreover, the variance *t*^2^ value was used to determine each study weight. Through the examination of homogeneity, the outcomes of aerobic capacity (homogeneity *p* value = 0.29), functional 6-min walking test (6MWT) (*p* value = 0.77), 10-m walk test (10MWT) (*p* value = 0.26), balance (*p* value = 0.48), steps (*p* value = 0.27), QOL (*p* value = 0.31) and cognition (*p* value = 0.55) are presented. For the demonstration of mean difference and CI between the experimental and control groups, multiple forest plots were run. Various meta-analyses were performed to evaluate the variables of interest.

## 3. Results

### 3.1. Study Selection

The preliminary literature search concluded with 7862 articles. Excluding the duplicates (*n* = 21), 7841 papers were viewed for their titles and abstracts. Of the 74 studies that underwent full examination, 11 RCTs passed the full eligibility criteria and entered this systematic review and meta-analysis.  Figure 1 explains the details of the search procedure.

Regarding the risk of bias, seven studies were considered to show some concerns [34–40], three presented low risk of bias [41–43] and one has high risk of bias [44]. As presented in Figure 2, the majority of the studies demonstrated some concerns of risk of bias on the D2 domain of the RoR2 tool.

### 3.2. Participants Characteristics

From the 11 included studies, 458 stroke survivors participated in the control or experimental group, where 64.8% were males (*n* = 297), 35.1% females (*n* = 161) and 0.1% of unknown gender (*n* = 2). The mean age of the participants was 62.14 (SD = 5.37) years old, and the mean stroke onset was 28.6 (SD = 21.87) months. Regarding the affected side of symptoms, 170 (37.1%) people present right hemiplegia, 179 (39%) with left hemiplegia and 15 (3.2%) had bilateral symptoms. The affected side was not mentioned in two studies: Ivey et al. [30] and Lapointe et al. [34, 38]. In addition, the type of stroke was not mentioned in the studies of Ivey et al. and Munari et al. [38, 39]. There was homogeneity found in the sample characteristics including number of participants, male, female and age (*p*=0.70, *p*=0.58, *p*=0.51, *p*=0.55).  Table 1 presents the demographic results of each included study.

### 3.3. Intervention Characteristics

For the experimental group, all 11 included RCTs applied HIIT programs. A 33% of the studies (*n* = 4) provided 36 total sessions with frequency: three times per week for 63% of the studies (*n* = 7). Two studies (18%) did not provide information about the total sessions [35, 38]. The duration of intervention ranged from 24 to 50 min, but it was not mentioned in the study of Lapointe et al. [34]. For the control group, four studies applied interval training of moderate intensity [34, 41–43], two studies of low intensity [38, 39], four studies offered conventional physiotherapy [34–36, 44] and two studies educational aerobic programs [37, 40] (Table 2). Samples' intervention characteristics including frequency, duration and number of sessions presented homogeneity (*p*=0.70, *p*=0.38, *p*=0.74, respectively).

### 3.4. Aerobic Capacity: VO_2_ Peak

**Figure 3:**
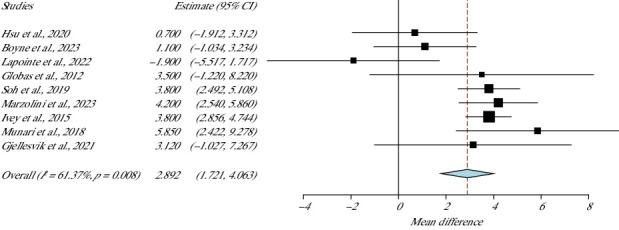
Results of the VO_2_ peak meta-analysis.

For the aerobic capacity, nine studies, with a total of 369 participants, measured VO_2_ peak and were included in the meta-analysis to examine the effect sizes of their interventions [34, 36, 38–44]. As shown in Figure 3, moderate heterogeneity of 61.3% was presented (*I*^2^ = 61.37%, *p* value Het = 0.008) with statistically significant differences in favour of the experimental group that used HIIT program (*p* value = 0.001, 95% CI: 1.72–4.06).  Table 3 shows all the results of the included studies.

### 3.5. Aerobic Capacity: HR

**Figure 4:**
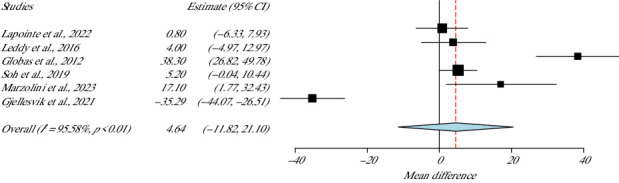
Results of the HR meta-analysis.

Six studies measured the HR of 243 participants [34–36, 40, 41, 44] demonstrating high heterogeneity (I^2^ = 95.5%, *p* value Het < 0.001), with no statistical difference between the groups (*p* value = 0.58, 95% CI = −11.82–21.10) as presented in Figure 4.

### 3.6. Functionality: 6-min Walking Test (6MWT)

**Figure 5:**
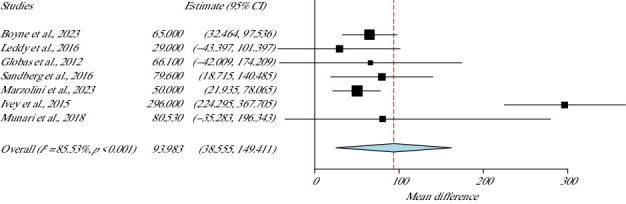
A 6-min walking test meta-analysis.

To assess the motor function of the participants, seven studies applied the 6MWT [35–39, 41, 42] to a total of 277 participants. Meta-analysis (Figure 5) showed high heterogeneity (*I*^2^ = 85.5%, *p* value Het < 0.001), where statistically significant differences in favour of the groups that applied HIIT (*p* value < 0.001, 95% CI = 38.55–149.41) were found.

### 3.7. Functionality: 10-m Walking Test (10MWT)

**Figure 6:**
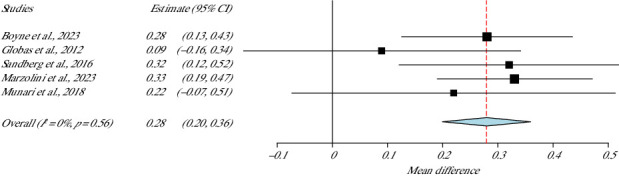
10-m walking test meta-analysis.

Five studies used the 10MWT to measure motor function of the participants [36, 37, 39, 41, 42]. In these studies, a total of 210 stroke survivors were included, presenting statistical importance difference between the groups in favour of the HIIT group (*p* value < 0.01, 95% CI = 0.20–0.36). This meta-analysis showed zero heterogeneity (*I*^2^ = 0%, *p* value Het = 0.56) (Figure 6).

### 3.8. Functionality: Time Up and Go (TUG)

**Figure 7:**
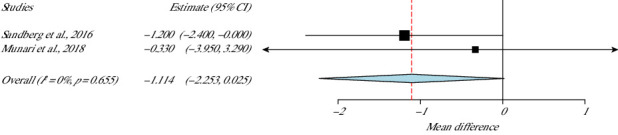
TUG meta-analysis.

Only two studies used TUG to evaluate motor and balance capacity of their 72 total participants [37, 39]. This meta-analysis had zero heterogeneity (*I*^2^ = 0%, *p* value Het = 0.65) as shown in Figure 7. Based on the data, no statistical differences were displayed between the experimental and the control groups (*p* value = 0.055, 95% CI = −2.25 to 0.02).

### 3.9. Functionality: Berg Balance Scale (BBS)

**Figure 8:**
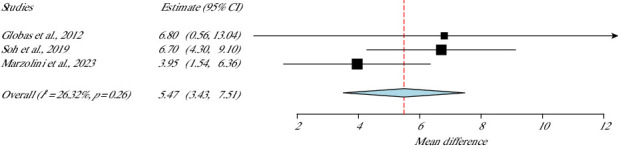
BBS meta-analysis.

For the examination of the balance of their 119 stroke survivors, three studies had used BBS [32, 37, 40] and presented statistical improvement in favour of the experimental group (*p* value < 0.01, 95% CI = 3.43–7.51). This meta-analysis showed low heterogeneity (*I*^2^ = 26.32 *p* value Het = 0.26) as shown in Figure 8.

## 4. Gait: Step Count

**Figure 9:**
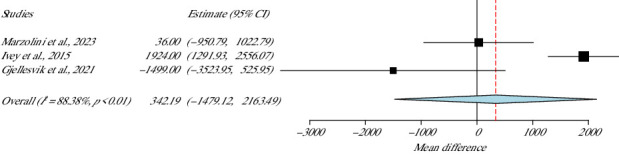
Step count meta-analysis.

Three studies, including 151 participants, examined gait by measuring the total steps that each participant had taken [38, 40, 41]. There was no statistical difference between the groups (*p* value = 0.71, 95% CI = −1479–2163) and high heterogeneity existed in this meta-analysis (*I*^2^ = 88.36%, *p* value Het < 0.01) (Figure 9).

### 4.1. QOL: EQ-5D

**Figure 10:**
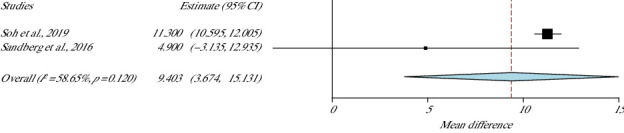
EQ-5D meta-analysis.

The EQ-5D tool was used in two studies [37, 44] to examine QOL of 92 people. Although the two studies showed moderate *h* (*I*^2^ = 58.6%, *p* value Het = 0.12), with varying levels of enhancement, the pooled analysis presented statistically significant positive effect for the QOL (*p* value = 0.001, 95% CI = 3.67–15.13) in favour of the HIIT group (Figure 10).

### 4.2. Cognitive Function: Montreal Cognitive Assessment (MoCA)

**Figure 11:**
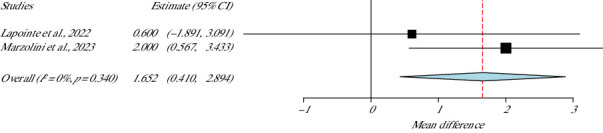
MoCA meta-analysis.

For the evaluation of cognitive function, two studies applied the tool MoCA [41, 45], including 73 stroke survivors. The heterogeneity was null with insignificant difference (*I*^2^ = 0%, *p* value Het = 0.34). The pooled means difference from this meta-analysis test was escorted by 95% CI = 0.41 to 2.89, indicating statistically significant differences (*p* value = 0.009) in favour of the experimental group, even with the lower bound (95% CI = 0.41) recommending small meaningful improvement regarding the cognitive function of the participants, as shown in Figure 11.

## 5. Discussion

This systematic review and meta-analysis evaluated and presented the impact of HIIT program, resulting in significant improvement of the aerobic capacity, motor and cognitive functionality and QOL for poststroke survivors. During the systematic search of this review, 11 RCTs [34–44] met the inclusion criteria and passed the homogeneity tests and were therefore included in the meta-analysis, extracting 458 participants in total.

All studies applied baseline and postintervention evaluations, with four having applied follow-up evaluations. More specifically, one study used a 4-week follow-up [44], the study of Lapointe et al. a 6-month follow-up [34] and two studies applied a 1-year follow-up [36, 40].

Various long-term limitations are presented in stroke survivors, as 40% of poststroke people are dependent on others for the implementation of ADL, due to physical, motor and cognitive symptoms [46].

Based on the general bibliography, it is known that CVA affects the cognitive function of stroke survivors, including the memory, attention and executive function of stroke survivors, where in the first year of the onset of stroke, a percentage of 30% of people develop vascular dementia [47]. Multiple physical activities such aerobic exercises, strength and balance exercises were applied for the evaluation of cognition, leading to an improvement of attention, especially under the application of combined training [48]. In this review, the study of Hsu et al. used the tool MMSE, delivered nonstatistical improvement on cognition [43], where two studies evaluated the cognition of stroke survivors via MoCA, providing positive impact of HIIT on cognitive function [41, 45]. HIIT advances motor skills and memory function, promoting brain-derived neurotrophic factor levels and neuroplasticity, promoting cognition and brain health [49, 50]. Cardiovascular exercises promote neuroplasticity through the activation of primary motor cortex, improving cognition and motor learning [28]. On the contrary, some studies mentioned that acute exercises with moderate intensity are more beneficial on cognitive function [51, 52]. Therefore, the evaluation of exercises protocol in the future is essential, thus the improvement of cognition is positively correlated with the implementation of ADL [53, 54].

The execution of physical exercises and ADL is limited because of restrictions on mobility, coordination and balance, resulting in physical inactivity. Those limitations lead to 50% reduction of cardiorespiratory function [12, 23, 46], deteriorating the general health of stroke survivors [55].

The increase recruitment of motor units of the CNS, the blood flow, the maximal cardiac output and the increase stroke volume leads to adaptations in endurance capacity, increasing VO_2_ max, under the use of HIIT [56]. Nine studies examined aerobic capacity with VO_2_ peak (with cardiorespiratory exercise testing) [34, 36, 38–44] leading to significant improvements. The included studies resulted in 2.8 mL/kg/min, which corresponds to an increase of 0.8 MET, following a reduction of 8% in cardiovascular mortality [57]. The majority of the studies found the effectiveness of HIIT in stroke survivors' rehabilitation for their aerobic capacity, which is consistent with studies in the literature [58, 59]. In contrast, the study of Boyne et al. (2023) presented with no such difference [60]. The results indicated major neuromotor limitation of participants with stroke in comparison to their aerobic capacity, using an average HR reverse (HRR) up to 60%, where in this systematic review, the majority of the included studies applied an experimental intervention of up to 80% of HRR [34, 36, 37, 39, 41, 43, 44]. HIIT increases mitochondrial biogenesis and Ca^2+^ uptake into the sarcoplasmic reticulum, resulting in better skeletal muscle function and decreased fatigue [28].

Only two studies compared HIIT to usual care [34, 40] and found a significant increase in aerobic capacity for the experimental group. This demonstrates the importance of HIIT and physical training for the improvement of metabolic stress and cardiovascular adaptations, leading to the enhancement of aerobic capacity [27, 58].

For the evaluation of motor function of people with CVA, different clinical tools were used. Seven studies used the 6MWT [35–39, 41, 42], and overall different results were found. The study of Marzolini et al. [41], where the participants used assistive device during the test, including quad cane and rollers, reported improvements but with no statistical differences between HIIT and moderate-intensity continuous training (MICT) after 24 weeks [41]. On the other hand, the study of Boyne et al. [42] showed positive results in favour of HIIT, where the stroke survivors used assistive ambulatory devices like single and quad cane [42]. Therefore, the type of assistive device used during the gait affects the total covered distance of 6MWT, as reported in Allet et al. [62], where the use of a single cane increased the walking distance by 15 m [62, 63].

Furthermore, the longitudinal study of Boyne et al. showed that high-intensity aerobic training in combination with gait training, when implemented with fastest walking speed, seems to increase motor function and gait parameters [60]. In accordance with this study, just two of the included reviewed papers applied high-intensity aerobic protocols in comparison to conventional physiotherapy, leading to increase of gait speed, examined with the 10MWT [36, 37]. In opposition, three studies compared HIIT to other aerobic training, demonstrating no significant improvement on gait speed [39, 41, 42]. Following the literature, important is the adoption of HIIT programs for the rehabilitation of poststroke people, as HIIT appears to enhance motor and gait characteristics, especially for gait speed in comparison to usual treatment [21, 64].

Due to motor limitations and neurological signs, 48.1% of stroke survivors present balance impairments [65]. One commonly used scale that is applied to examine balance in poststroke people is BBS, presenting high internal consistency (Cronbach alpha = 0.92–0.98) [66]. In this systematic review, homophonous was the positive impact of HIIT in balance, after the examination of the three studies that used BBS [36, 41, 44]. The need of repetitive body positioning and direction changes through HIIT is common, resulting in balance improvements [67]. The studies that applied HIIT in comparison to the conventional training showed that HIIT provides positive results on balance [36, 44], similarly to the study of Gjellesvik et al., stating that HIIT combined with standard care is superior to conventional intervention for balance and function in this population [68].

Only two studies used the TUG test [37, 39], where this meta-analysis showed that HIIT was no superior to other interventions for motor function and falls. The results matched the literature results, presenting low to strong correlations between TUG and step count [69–71]. Regarding total amount of steps, only three studies measured the mentioned outcome [38, 40, 41] concluding to similar effects to TUG. The parameters of gait and the increased number of steps affects the time of completion of TUG [70, 71]. In addition to the total amount of steps, TUG is dependent on the turning task of the test, motor and/or cognitive dual tasking activities [70].

CVA affects multiple health domains, causing ADL and QOL limitations for poststroke survivors. Multiple clinical tools can be used to examine health-related quality of life (HRQoL), where a strong and valid tool for HRQoL assessment is the EQ-5D [72–74]. However, only two studies assessed QOL using the EQ-5D [37, 44]. Both studies compare HIIT to conventional training, finding absolute positive effect in favour of HIIT. Considering the small number of studies, it is important to note with caution that the results of our meta-analysis are in agreement with the literature, reporting that motor and cardiorespiratory improvements lead to QOL enhancement [75, 76].

In conclusion, this systematic review and meta-analysis report important findings regarding the efficacy of HIIT following a stroke episode. HIIT is an alternative rehabilitation solution used to optimize cardiovascular exercises with time-efficient approach, leading to health improvements for stroke survivors [27]. HIIT is essential to be applied during the first weeks after a CVA in order to promote neuroplasticity, motor function, including balance and gait characteristics [58, 76] and prevent cardiovascular and general health deterioration [50].

## 6. Limitations

Some research bias concerns have been noted in 7 of the 11 studies included in this systematic review. In particular, two studies showed mediate bias due to missing data that could affect accuracy and validity of the results [38, 44]. Absence of double blinding in the majority of studies [34–40, 44] was a methodological restriction, and it is important to contemplate during the designing of future studies. This review ran and demonstrated results from various meta-analysis tests based on small number of studies. The use of different assessment tools and the small number of data and participants in some tests resulted in limiting the development of more data-driven meta-analysis for the provision of more comprehensive information on the specific topic. Although the included studies demonstrated interesting findings with regard to the beneficial effects of HIIT for poststroke survivors, the different types of interventions lead to an unclear picture.

## 7. Conclusion

Concluding the results of this systematic review and meta-analysis, HIIT is an efficient approach for the development of rehabilitation programs for poststroke individuals. However, small number of RCTs examined the effectiveness of HIIT programs in stroke; therefore, more studies should be conducted in the future. Considering the steady increase of CVA rates, the development of appropriate protocols for stroke survivors is essential, giving the opportunity to evaluate and offer safe and valid information to the clinical and general population.

## Figures and Tables

**Table 1:** Sample characteristics per study.

Study	Number of participants	Gender	Age (SD)	Stroke onset (months) (SD)	Type of stroke	Affected symptomatic side
Boyne et al. [42]	55	Male = 36 (65.4%) Female = 19 (34.6%)	62.65 (9.9)	30 (15.6)	IS = 34 HS = 21	Right = 27 (49%) Left = 28 (51%)
Marzolini et al. [41]	47	Male = 38 (80.8%) Female = 9 (19.2%)	61.85 (10.6)	10.7 (8.4)	IS = 36 HS = 11	Right = 22 (47%) Left = 19 (40%) Bilateral = 6 (13%)
Lapointe et al. [34]	52	Male = 33 (63.4%) Female = 19 (36.6%)	69.2 (10.7)	39.2 (61)	IS = 52	N/A
Gjellesvik et al. [40]	70	Male = 29 (41.4%) Female = 41 (58.6%)	57.9 (9.2)	26.4 (14.6)	IS = 57 HS = 13	Right = 25 (36%) Left = 34 (49%) Bilateral = 11 (15%)
Hsu et al. [43]	23	Male = 20 (87%) Female = 3 (13%)	55.8 (N/A)	33.6 (N/A)	IS = 15 HS = 8	Right = 12 (52%) Left = 11 (48%)
Soh et al. [44]	36	Male = 25 (69.4%) Female = 11 (30.6%)	56.85 (6.25)	6.2 (5.15)	IS = 25 HS = 11	Right = 13 (33%) Left = 23 (67%)
Munari et al. [39]	16	Male = 15 (93.6%) Female = 1 (6.2%)	61.5 (8.52)	60 (40.14)	N/A	Right = 9 (56%) Left = 7 (44%)
Leddy et al. 2016 [35]	33	Male = 23 (69.6%) Female = 10 (31.4%)	56.33 (12)	3.5 (4)	IS = 25 HS = 8	Right = 11 (33%) Left = 22 (67%)
Sandberg et al. [37]	56	Male = 28 (50%) Female = 28 (50%)	70.85 (7.55)	1 (10.3)	IS = 55 HS = 1	Right = 28 (50%) Left = 24 (43%) Bilateral = 2 (7%)
Ivey et al. [38]	34	Male = 21 (61.7%) Female = 13 (38.3%)	62 (1.5)	39 (13)	N/A	N/A
Globas et al. [36]	36	Male = 29 (80.5%) Female = 7 (19.5%)	68.65 (6.3)	65.1 (57.3)	IS = 36	Right = 23 (67%) Left = 13 (33%)

*Note:* N/A = not available.

Abbreviations: HS = haemorrhagic stroke, IS = ischaemic stroke.

**Table 2:** Intervention characteristics per study.

Study	Participants (*n*): experimental group/control group	Frequency (times/week)	Duration (minutes)	Total sessions	HIIT intervention	Control group intervention
Boyne et al. [42]	*N* = 56 EG = 28 (50%) MICT = 28 (50%)	3	40	36	Training > 60% HRR: 3 min warm up, 10 min overground training, 20 min treadmill walking, 10 min overground training, 2 min cool down	Continuous walking 40–60% HRR
Marzolini et al. [41]	*N* = 47 EG = 24 (51%) MICT = 23 (49%)	5	30	120	5 min warm up, 20 min treadmill HIIT exs, 5 min cool down	MIE on treadmill 60 min
Lapointe et al. [34]	*N* = 52 EG = 19 (36.5%) MICT = 16 (45.7%) CPT = 17 (32.6%)	3	N/A	36	HIIT exs 20–40 min and 30 min unsupervised MICT home aerobic ex,	Ergocycle 20–40 min, 30 min of aerobic exs at home (walking, swimming, dancing, or cycling), and usual care
Gjellesvik et al. [40]	*N* = 70 EG = 36 (51.4%) EAP = 34 (48.6%)	3	38	24	10 min warm up, 4 × 4 min HIIT on treadmill and 3 min break	Usual care and education
Hsu et al. [43]	*n* = 23 EG = 10 (43%) MICT = 13 (57%)	3	30	18	Bicycle ergometer five x 3 min exercises (80% VO_2_ peak), 3 min exercises (40% VO_2_ peak), cool down	Ergocycle warm up, 3 min 60% VO_2_ peak
Soh et al. [44]	*N* = 36 EG = 18 (50%) CPT = 18 (50%)	2	50	28	High intensity skater exs 30 min	Conventional aerobic 30 min treadmill
Munari et al. [39]	*N* = 16 EG = 8 (50%) LOIT = 8 (50%)	3	50	36	50 min: 10 min warmup, 5 × 5 HIIT in treadmill, 3 min breaks and 5 min cool down (80–90% HRmax)	10 min wam up, treadmill LOIT treadmill, 5 min cool down
Sandberg et al. [37]	*N* = 56 EG = 28 (50%) EAP = 28 (50%)	3	30	36	Aerobic exs (15 min warm up, 8 min HIIT ergocycle exs, 10 min LOIT exs, 8 min HIIT ergocycle exs, 15 min cool down)	Physical activity education
Leddy et al. [35]	*N* = 33 EG = 21 (63.6%) CPT = 12 (36.3%)	3	40	N/A	High intensity stepping exs 40 min, treadmill walking training	Active lower extremity exs, stretching exs, transfers, balance activities, gait, stairs
Ivey et al. [38]	*N* = 34 EG = 18 (50%) LOIT = 16 (50%)	N/A	30	N/A	HIIT treadmill	LOIT treadmill
Globas et al. [36]	*N* = 36 EG = 18 (50%) CPT = 18 (50%)	4	40	40	HIIT treadmill exs 30–50 min	Conventional physiotherapy (passive, ULs & LLs muscle tone regulating exs with elements of balance exs)

*Note:* LOIT: low-intensity training, N/A: not available, Exs: exercise.

Abbreviations: CPT, conventional physiotherapy; EAP, educational aerobic program; EG, experimental group; MICT, moderate-intensity continuous training; MIE, moderate-intensity exercise.

**Table 3:** Results of meta-analysis.

**Studies**	**Weight (%)**	**V** **O**_2_ **peak**

Boyne et al., 2023	12.6	2.892 (1.721–4.063)
Marzolini et al., 2023	15.1	
Lapointe et al., 2022	7.0	SE = 0.598
Gjellesvik et al., 2021	5.8	
Hsu et al., 2020	10.4	*p* value < 0.001
Soh et al., 2019	17.1	
Munari et al., 2018	7.6	
Ivey et al., 2015	19.1	
Globas et al., 2012	4.8	

**Studies**	**Weight (%)**	**HR**

Marzolini et al., 2023	15.37	4.64 (−11.82–21.10)
Lapointe et al., 2022	17.16	
Gjellesvik et al., 2021	16.88	SE = 8.40
Soh et al., 2019	17.42	
Leddy et al., 2016	16.85	*p* value = 0.58
Globas et al., 2012	16.32	

**Studies**	**Weight (%)**	**6MWT**

Boyne et al., 2023	17.5	93.983 (38.555–149.411)
Marzolini et al., 2023	17.7	
Munari et al., 2018	10.2	SE = 28.280
Leddy et al., 2016	14.1	
Sandberg et al., 2016	15.2	
Ivey et al., 2015	14.2	*p*value < 0.001
Globas et al., 2012	10.9	

**Studies**	**Weight (%)**	**10MWT**

Boyne et al., 2023	28.81	0.28 (0.20–0.36)
Marzolini et al., 2023	34.76	
Munari et al., 2018	8.05	SE = 0.04
Sandberg et al., 2016	17.46	
Globas et al., 2012	10.92	*p* value < 0.01

**Studies**	**Weight (%)**	**BBS**

Marzolini et al., 2023	45.06	5.47 (3.43–7.51)
Soh et al., 2019	45.16	SE = 1.04
Globas et al., 2012	9.78	*p*value < 0.01

**Studies**	**Weight (%)**	**TUG**

Munari et al., 2018	9.8	−1.114 (−2.253 to 0.025)
Sandberg et al., 2016	90.1	SE = 0.581
		*p*value = 0.055

**Studies**	**Weight (%)**	**Step count**

Marzolini et al., 2023	35.53	342.19 (−1479.1–2163.4)
Gjellesvik et al., 2021	26.62	SE = 929.26
Ivey et al., 2015	37.86	*p*value = 0.71

**Studies**	**Weight (%)**	**EQ-5D**

Soh et al., 2019	70.3	9.403 (3.674–15.131)
Sandberg et al., 2016	29.6	SE = 2.923
		*p* value = 0.001

**Studies**	**Weight (%)**	**MoCA**

Marzolini et al., 2023	75.13	1.652 (0.410–2.894)
Lapointe et al., 2022	24.86	SE = 0.634
		*p* value = 0.009

*Note:* VO_2_ peak: peak oxygen uptake, 6 MW: 6 min walking test, 10MWT: 10 m walk test, TUG: Time Up and GoEQ-5D: Euroqol 5 Dimensions, MoCA: Montreal Cognitive Assessment.

Abbreviations: BBS, Berg balance scale; HR, heart rate.
